# Solubility of sulfur dioxide in tetraglyme-NH_4_SCN ionic liquid: high absorption efficiency†

**DOI:** 10.1039/c8ra08991d

**Published:** 2018-12-18

**Authors:** Qiang Xu, Wei Jiang, Jianbai Xiao, Xionghui Wei

**Affiliations:** College of Chemistry and Molecular Engineering, Peking University 100871 PR China xhwei@pku.edu.cn

## Abstract

An easily prepared ionic liquid was synthesized by a one-step method and applied in SO_2_ absorption efficiently. The cation of the ionic liquid is a supramolecular structure consisting of NH^+^_4_ and tetraglyme, similar to the structure of NH^+^_4_ and crown ether, and the anion is selected as SCN^−^. The ionic liquid has good thermal stability. Under the conditions of 293 K and 1 bar, one mol ionic liquid can absorb 2.73 mol SO_2_, which is about 30% higher than tetraglyme. The absorption mechanism was characterized using IR and NMR. And the results confirmed that the interaction mechanism between SO_2_ and the ionic liquid is a physical interaction rather than a chemical interaction.

## Abstract 2

Simple prepared tetraglyme-NH_4_SCN ionic liquid is used to absorb sulfur dioxide efficiently and regenerate.

## Graphical abstract

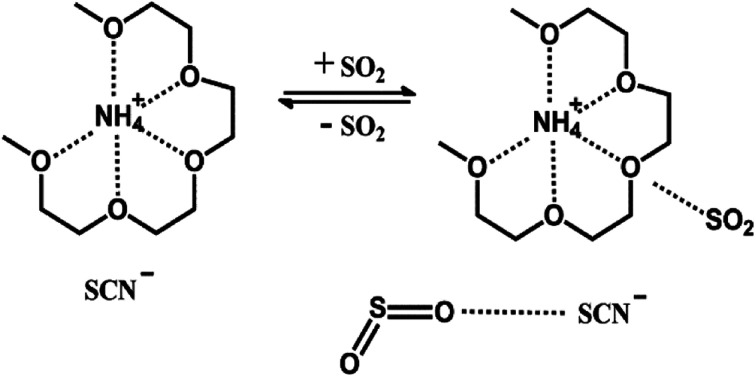

## 1. Introduction

Sulfur dioxide (SO_2_) is a common atmospheric pollutant mainly derived from the burning of fossil fuels.^1^ SO_2_ emitted from flue gas will cause serious harm to the natural environment^2^ and human health.^3^ In recent years, the theme of removing SO_2_ gas from flue gas has attracted widespread attention around the world. At present, a limestone/lime based FGD method is the most widely used flue gas desulfurization technology in industrial practice.^4,5^ However, this method produces a large amount of secondary pollution such as gypsum and industrial wastewater, along with low limestone utilization and poor selectivity, which is difficult to overcome.^6^ Therefore, the development of new desulfurizers with good absorption capacity, selectivity, regenerability, thermal stability and an environmentally friendly nature has become one of the current research hotspots.

Recently, ionic liquids (ILs), as new candidate solvents to absorb SO_2_ in flue gas, have been widely studied due to their low saturated vapor pressure,^7^ high thermal and chemical stability, and excellent solubility to some substances.^8^ The ionic liquids used for SO_2_ absorption mainly include the following types: guanidinium ionic liquid,^9–13^ hydroxyl ammonium ionic liquid,^14–16^ imidazolium ionic liquid,^17–21^ tetrabutyl ammonium ionic liquid^22–24^ and quaternary phosphine ionic liquids.^25^ However, these ionic liquids are relatively weak in their ability to absorb SO_2_. One reason is that they were not originally designed to absorb SO_2_ and therefore are not optimized for SO_2_ absorption. The ability of an ionic liquid to absorb SO_2_ is closely related to the type of cation and anion of the ionic liquid itself. For example, the absorption capacity of [C_4_Py][BF_4_] ionic liquid at 293 K and 0.1 MPa is 0.440 g g^−1^ ionic liquid, which is better than that of [C_8_Py][BF_4_] (0.378 g g^−1^ ionic liquid) but weaker than [C_4_Py][SCN] (0.841 g g^−1^ ionic liquid).^26^

In order to improve the absorption capacity of ionic liquids, researchers prepared a variety of functional ionic liquids. There are two main types of functional ionic liquids, including ether functional ionic liquids^27–31^ and amine functional ionic liquids.^32–34^ The latter generally has poor selectivity and desorption ability due to its chemical interaction with SO_2_. In contrast, the former have better absorption ability and superior selectivity than those of the original ionic liquids due to the physical interaction between the ether functional groups and SO_2_.

As a functional group, ether groups, mainly referred to herein as ethylene glycol and its derivatives, have good absorption capacity and good regenerability for SO_2_, thus giving it a wide range of potential industrial applications.^35,36^ However, ethylene glycol and its derivatives alone have two disadvantages that are difficult to overcome. One is its less prominent SO_2_ absorption capacity and the other is its relatively high vapor pressure. The latter causes it to be more volatile during the absorption–regeneration process, which in turn leads to excessive solvent loss. The introduction of ethylene glycol and its derivatives into ionic liquids could effectively solve the above two problems. As functional groups in the ether-based ionic liquid, ethylene glycol and its derivatives make the ether-based ionic liquids have many of the advantages mentioned above in absorbing SO_2_.

Conventional ether functional ionic liquids are difficult to industrialize due to their complicated synthesis and high cost. A simple one-step method by mixing and stirring was developed for ionic liquid synthesis. And this type ionic liquids have been used in nonaqueous electrolytes in Li batteries^37,38^ and CO_2_ absorption.^39^ This method is dedicated to solving the problem that conventional ionic liquids are difficult to synthesize. We have previously prepared a series of glycine-lithium salt ionic liquids and studied their absorption of SO_2_.^40^ These readily synthesized glyme-lithium salt ionic liquids have a greater thermal stability than glymes while maintaining similar SO_2_ absorption capabilities. However, the higher price of lithium salt leads to higher cost of preparation of the corresponding ionic liquid, which severely limits the application of the ionic liquid in desulfurization. In order to solve this problem and further improve the absorption capacity of ionic liquids for SO_2_, a novel ether-based ionic liquid with advantages of simple synthesis, strong absorption capacity and low cost has been developed.

**Fig. 1:**
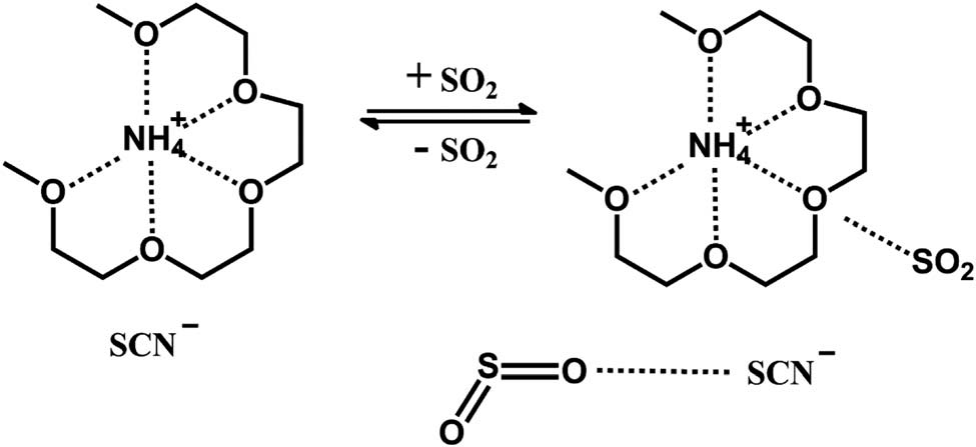
Structure of [NH^+^_4_-tetraglyme][SCN] ionic liquid before and after absorbing SO_2_.

This ionic liquid was synthesized by tetraglyme (G4) and NH_4_SCN, and its structure is shown in Fig. 1. In order to increase the SO_2_ absorption capacity of the ionic liquid, SCN^−^ was chosen as the anion. The absorption and desorption performance of the ionic liquid were investigated. The interaction mechanism between the ionic liquid and SO_2_ was also studied by infrared spectroscopy (IR) and nuclear magnetic resonance (NMR).

## 2. Experimental section

### 2.1 Chemicals

Tetraglyme (AR) was purchased from Shanghai Aladdin Bio-Chem Technology Co., LTD, and NH_4_SCN (AR) was purchased from Sinopharm Chemical Reagent Co., Ltd. All reagents were used without further purification. Chromatographic grade ethanol and distilled water are also used for this work. Certified standard pure SO_2_ gas (>99.9%) and N_2_ (99.9% purity) supplied by Beijing Gas Centre, Peking University (China) is used to determine the SO_2_ absorption capacity of the ionic liquid.

### 2.2 Preparation of the ionic liquid

Tetraglyme and NH_4_SCN were mixed in a molar ratio of 1 : 1, and then the liquid mixture was stirred and heated at 303 K for 6 hours, so that [NH_4_-tetraglyme][SCN] ionic liquid can be obtained. The ionic liquid was then dried under vacuum for 48 hours at room temperature. The resulting ionic liquid is a clear, pale yellow liquid.

### 2.3 Absorption and desorption of SO_2_

The absorption and desorption experiments of SO_2_ were carried out in an absorption tube with an inner diameter of 15 mm. SO_2_ at a flow rate of 100 mL min^−1^ was bubbled through the absorbent tube containing absorber sample. The constant temperature required for absorption and regeneration is maintained by a circulating water bath into which the absorber tube is immersed. The absorption capacity of SO_2_ was determined by means of weighing. In the absorption experiments under different pressures, a mixed gas having different partial pressures of SO_2_ was obtained by controlling the flow rates of SO_2_ and N_2_. In the regeneration experiment, the temperature was maintained at 353 K, and the flow rate of nitrogen was 100 mL min^−1^. And the analytical method was similar to that of absorption.

## 3. Results and discussion

### 3.1 Properties of the ionic liquid

The conductivity of the ionic liquid was measured to be 1528 μS cm^−1^. In contrast, tetraglyme itself has a conductivity of 0. The viscosity of ionic liquid is 104 mPa s at 298 K, which is 31 times that of tetraglyme (3.295 mPa s (ref. 41)) at the same temperature.

The ionic liquid was characterized by MS, NMR and IR. The result of MS is shown in Fig. S1 in ESI.† It can be clearly seen that the ionic liquid has a cationic molecular weight of about 240.2, which is the sum of the molecular weight of tetraglyme and the molecular weight of ammonium ion. It is reasonably speculated that cation in ionic liquid should consist of an ammonium ion and a tetraglyme molecule with a structure similar to that of crown ether–ammonium ion, as shown in Fig. 1.

**Fig. 2:**
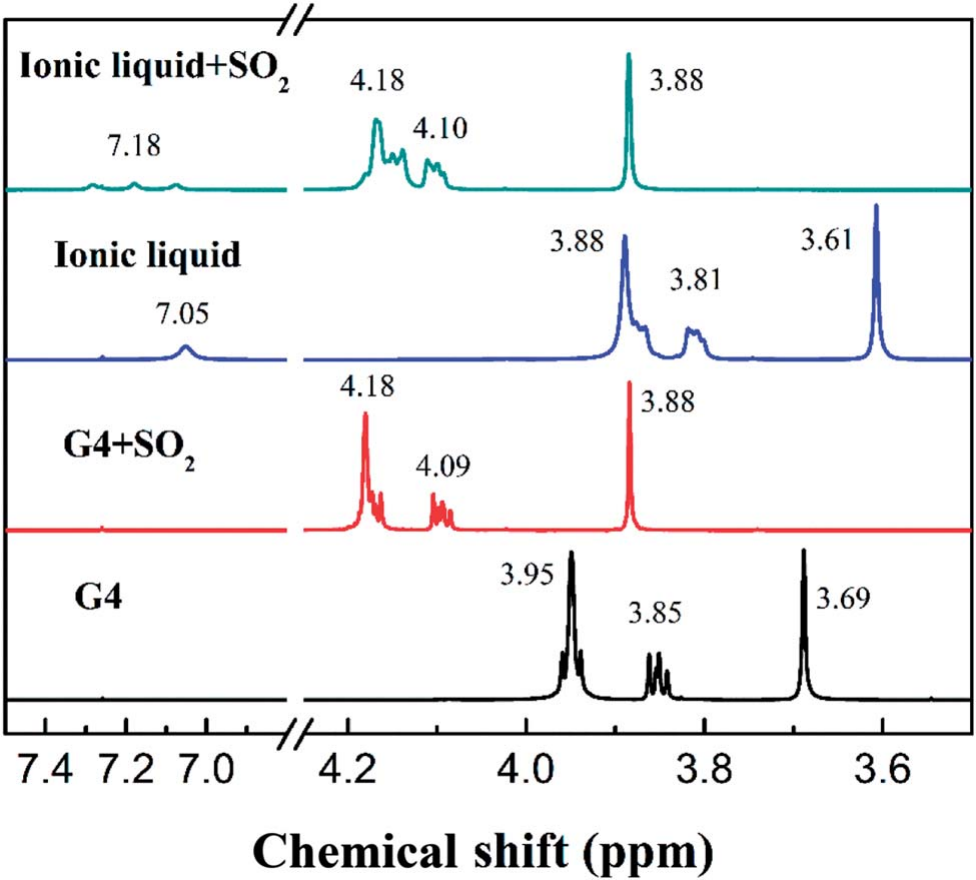
^1^H-NMR spectra of tetraglyme, tetraglyme after SO_2_ absorption, [NH^+^_4_-tetraglyme][SCN] and [NH^+^_4_-tetraglyme][SCN] after SO_2_ absorption, with CDCl_3_ as an external reference.

An external reference (CDCl_3_) method was used in ^1^H-NMR and ^13^C-NMR to avert the solvent effect by the deuterated reagents. The chemical shifts of the H atoms are shown in Fig. 2. It can be seen from the figure that the chemical shifts of the hydrogen atoms in the ether functional group move to the high field, and the chemical shifts change from 3.95, 3.85, and 3.69 of tetraglyme to 3.88, 3.81 and 3.61 of the ionic liquid, respectively. The main reason for the change in chemical shifts is that the deshielding effect caused by oxygen atoms is inhibited in virtue of the interaction between NH^+^_4_ and the oxygen atoms in tetraglyme group. The chemical shift of the H atom attached to the N atom appears at 7.05. Further, solvents having different molar ratio of tetraglyme and NH_4_SCN including 1 : 0.2, 1 : 0.4, 1 : 0.6 and 1 : 0.8, was prepared and the ^1^H-NMR spectra is shown in Fig. S2 in ESI.† According to the spectra, H atoms of NH^+^_4_ ions at different solvents above have similar chemical shifts, which means that the interaction strength between NH^+^_4_ ions and tetraglyme is close in several different solvents including the ionic liquid. The chemical shifts of the H atom in tetraglyme group also moves to the high field as the NH_4_SCN content increases in the solvents, which confirms the deshielding effect is inhibited in the ionic liquid.

**Fig. 3:**
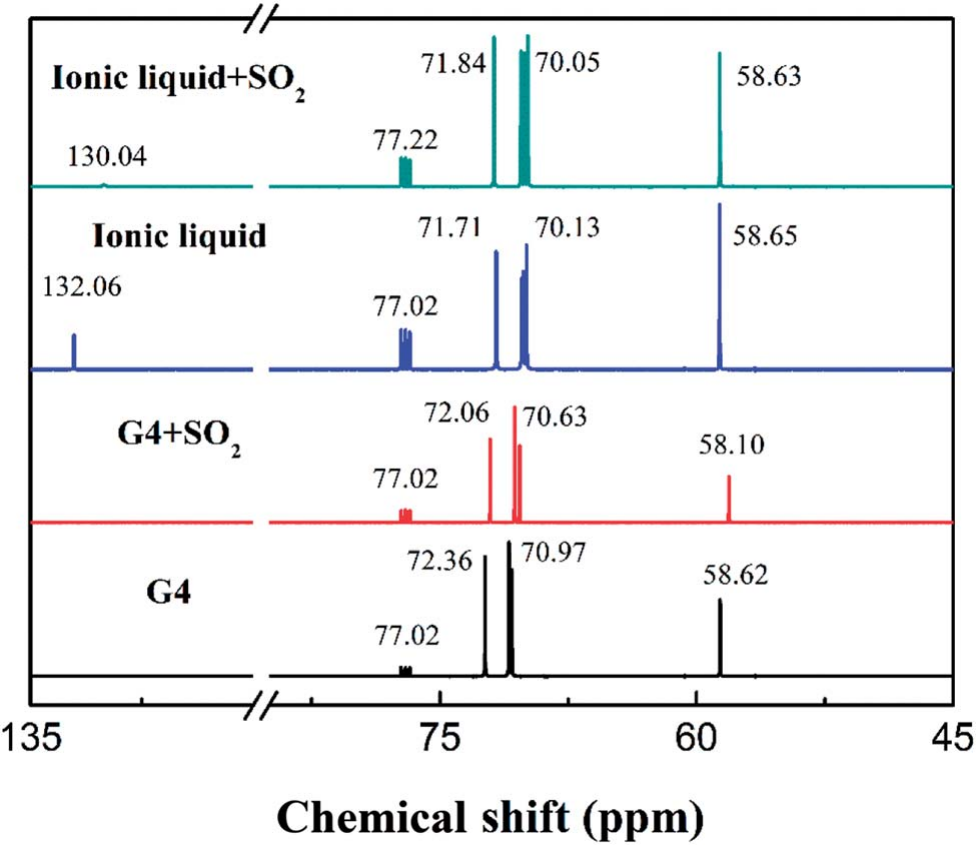
^13^C-NMR spectra of tetraglyme, tetraglyme after SO_2_ absorption, [NH^+^_4_-tetraglyme][SCN] and [NH^+^_4_-tetraglyme][SCN] after SO_2_ absorption, with CDCl_3_ as an external reference.

However, in the ^13^C-NMR spectrum shown in Fig. 3, there is no significant change in the chemical shifts of the tetraglyme carbon atoms after the formation of the ionic liquid. This indicates that there is no significant interaction between NH^+^_4_ and the carbon atoms of tetraglyme group in the ionic liquid. The existing ion–dipole interaction and hydrogen bonding between the two substances mainly occur between the hydrogen atoms of NH^+^_4_ and the oxygen atoms of tetraglyme.

**Fig. 4:**
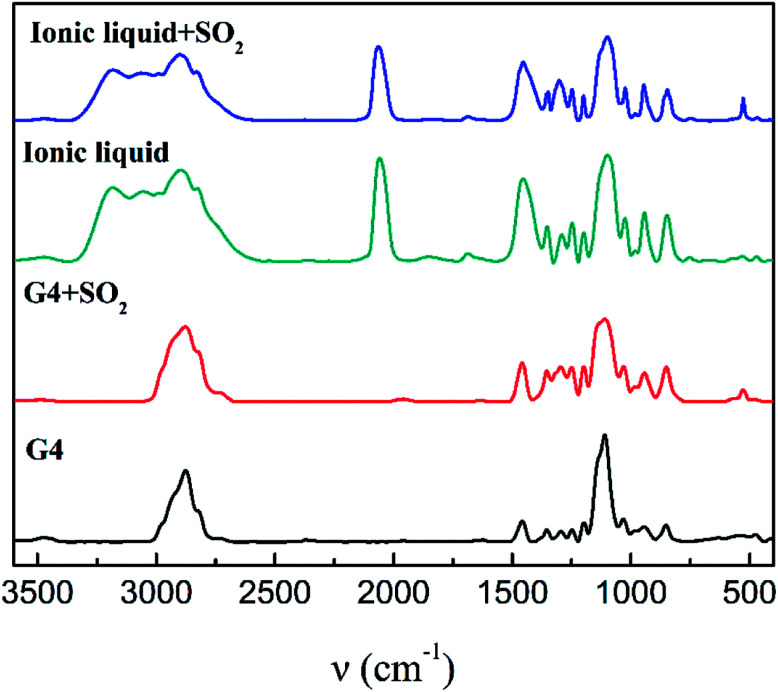
IR spectra of tetraglyme, tetraglyme after SO_2_ absorption, [NH^+^_4_-tetraglyme][SCN] and [NH^+^_4_-tetraglyme][SCN] after SO_2_ absorption.

Fig. 4 is an IR spectrum of tetraglyme and [NH_4_-tetraglyme][SCN] ionic liquid before and after SO_2_ absorption. There is no significant shift in the C–O vibration peak at 1110 cm^−1^ and the C–C vibration peak at 1430 cm^−1^, when tetraglyme forms an ionic liquid with NH_4_SCN. A closer comparison of the spectra of the two materials reveals another difference: the ionic liquid has a distinct absorption peak of SCN^−^ at 2064 cm^−1^. Meanwhile, tetraglyme has a C–H vibration peak at 2876 cm^−1^, but after the formation of ionic liquid, the displacement of this absorption peak changes significantly, which in turn produces a huge absorption peak between 2827 cm^−1^ and 3184 cm^−1^. This phenomenon indicates that after the formation of the ionic liquid, a very strong hydrogen bond is formed between the tetraglyme and the NH^+^_4_, resulting in a significant change in the position of the C–H bond in the tetraglyme molecular.

**Fig. 5:**
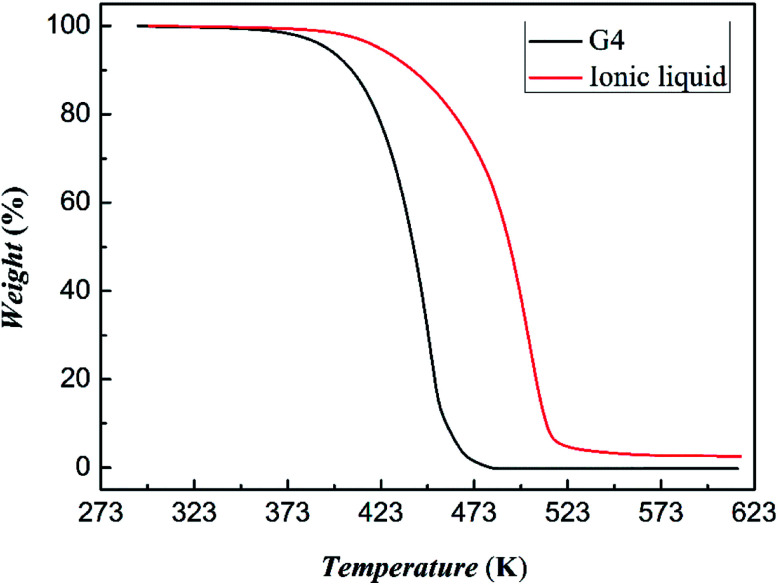
Thermal gravimetric analysis of tetraglyme and [NH^+^_4_-tetraglyme][SCN] ionic liquid.

The result of thermogravimetric analysis of tetraglyme and [NH_4_-tetraglyme][SCN] ionic liquid is shown in Fig. 5. It shows that the initial decomposition temperature of tetraglyme and the ionic liquid is 371 K and 389 K, respectively. It can be seen that the thermal stability of the [NH_4_-tetraglyme][SCN] ionic liquid is significantly better than that of tetraglyme itself. It was further noted that the temperature of the absorption experiment did not exceed 313 K, and the temperature of the desorption experiment was 353 K. This means that the normal operating temperature of the desulfurizer will generally not exceed 353 K. Therefore, we performed a constant temperature thermogravimetric experiment on [NH_4_-tetraglyme][SCN] ionic liquid and tetraglyme at 353 K. The results are shown in Fig. S3 in ESI.† As can be seen from the figure, tetraglyme has a higher volatility at 353 K, while the thermal stability of ether ionic liquid is rather low. This means that latter has satisfactory volatilization rate during the absorption–desorption process of the desulfurization experiment, avoiding excessive solvent loss in practical applications, thereby reducing the cost of desulfurization and avoiding environmental hazards caused by volatilization as much as possible.

Further thermal analysis experiments confirmed the strength of tetraglyme and NH_4_SCN, as shown in Fig. S4 in ESI.† According to DSC and TGA results, the interaction strength of tetraglyme and NH_4_SCN is about 4.03 kJ mol^−1^ at 373 K, which is close to the interaction strength between 15-crown-5 and NH_4_Cl.^42,43^

### 3.2 Absorption capacity of ionic liquid

**Fig. 6:**
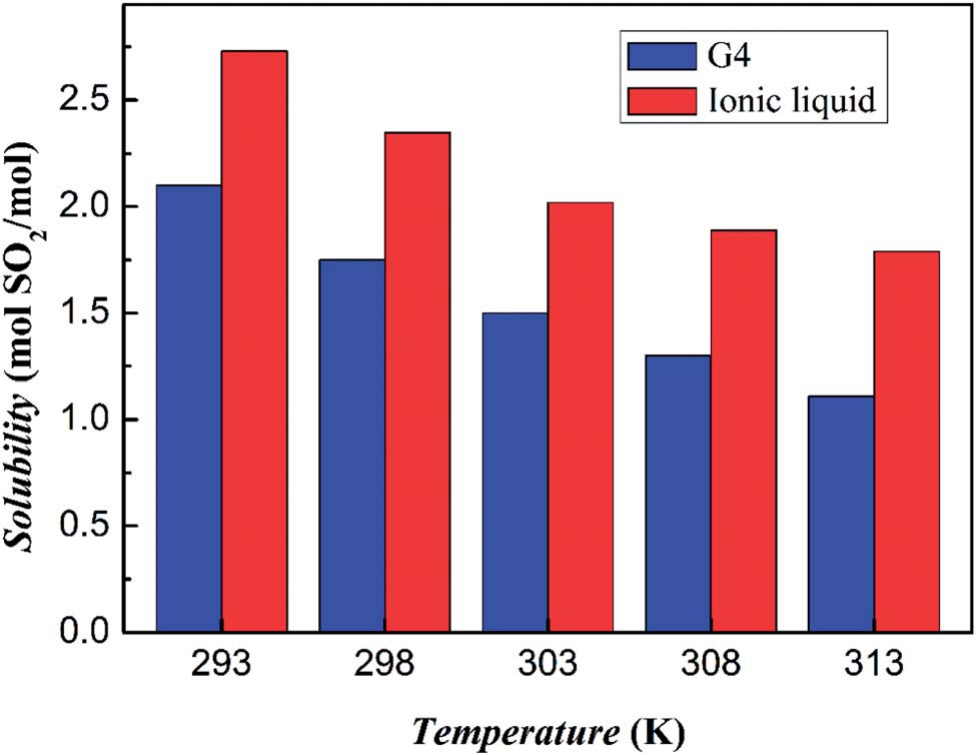
SO_2_ absorption capacities of tetraglyme and [NH^+^_4_-tetraglyme][SCN] at different temperatures with the pressure of SO_2_ equal to 1 bar.

The absorption capacity of SO_2_ at different temperatures and 1 bar is measured as shown in Fig. 6. It can be clearly seen that as the temperature increases, the absorption capacity of both the tetraglyme and the ionic liquid on the SO_2_ gas is decreased. However, at all temperatures studied, the ionic liquid has an absorption capacity that is about 30% higher than that of tetraglyme. At the condition of 293 K and 1 bar, 1 mol this ionic liquid can absorb about 2.73 mol of SO_2_ Correspondingly, the absorption capacity of tetraglyme is 2.10 mol. The result of the unit mass absorption capacity of tetraglyme and the ionic liquid is shown in Fig. S5 in ESI.† And it is apparent that the ionic liquid has a better unit mass absorption capacity than tetraglyme at the temperature exceeding 303 K.

**Fig. 7:**
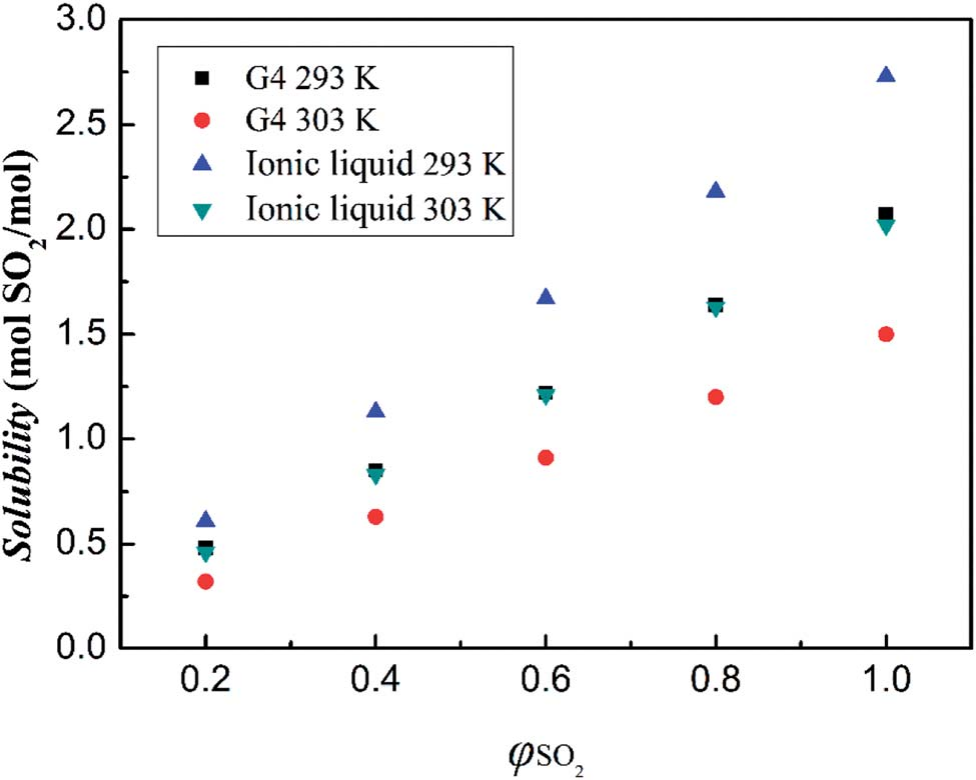
SO_2_ absorption capacities of tetraglyme and [NH^+^_4_-tetraglyme][SCN] at different SO_2_ partial pressures under the temperature of 293 K and 303 K.

The effect of SO_2_ partial pressure on the absorption of SO_2_ by ionic liquid has also been investigated. The results are shown in Fig. 7. As can be seen from the figure, when the SO_2_ volume fraction is increased from 20% to 100%, the absorption of the ionic liquid is increased from 0.61 mol SO_2_ pre mol ionic liquid to 2.73 mol SO_2_ pre mol ionic liquid at 293 K. It can be seen that with the increase of the volume fraction of SO_2_ gas, the absorption of SO_2_ by the ionic liquid gradually increases, and both the two variables have a linear relationship. This suggests that the absorption between the ionic liquid and SO_2_ should be dominated by physical interaction.

**Fig. 8:**
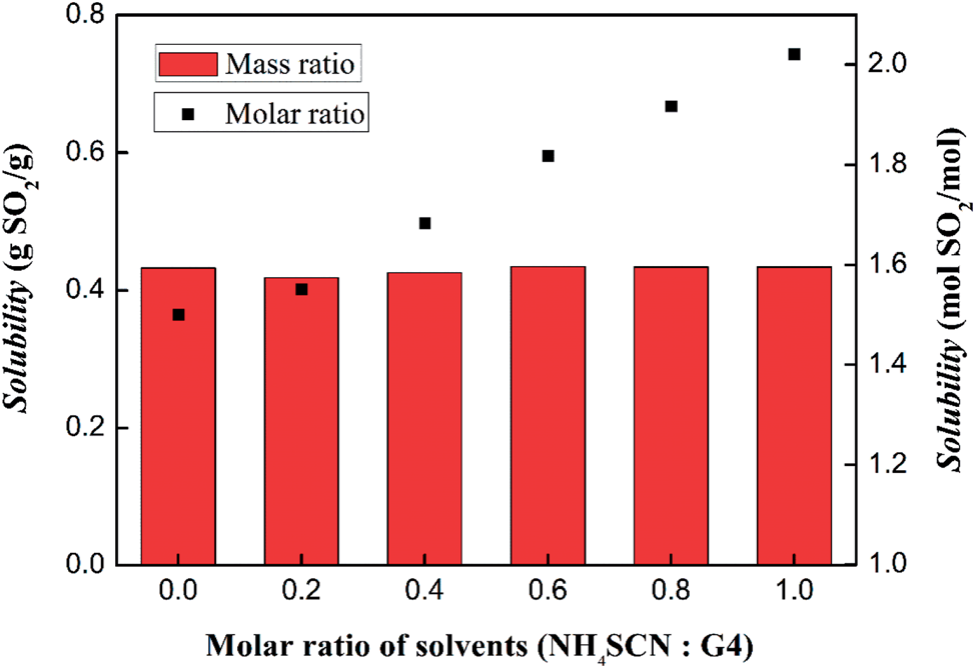
SO_2_ absorption capacities of solvents with different NH_4_SCN and tetraglyme molar ratio under the condition of 303 K and 1 bar.

The SO_2_ absorption capacities of solvents with different molar ratio of tetraglyme and NH_4_SCN were also test and the results are shown in Fig. 8. Under the condition of 303 K and 1 bar, the solvents have similar unit mass absorption from 0.41 g g^−1^ to 0.44 g g^−1^. However, it should be noted that the absorption per mole of solvent increased from 1.50 mol to 2.02 mol, as the NH_4_SCN content increases from 1 : 0.2 to 1 : 1. The increase of molar absorption is presumed to originate from the influence of SCN^−^ in the solvents.

### 3.3 Regeneration

Based on these behaviors of the ionic liquid, after the ionic liquid absorbs SO_2_, it can be desorbed by heating, nitrogen stripping or vacuum depressurization. Here, the desorption ability of the ionic liquid after absorbing SO_2_ was studied by a heating with nitrogen stripping method. The results show that after five absorption–desorption cycles, the absorption of SO_2_ by [NH_4_-tetraglyme][SCN] ionic liquid still have 99% capacity of the initial absorption, and the desorption rate reaches 98% (see Fig. S6 in ESI†). It can be seen that the [NH_4_-tetraglyme][SCN] ether ionic liquid has good absorption and desorption properties at the same time, which makes the absorbing-stripping process possible. Apparently, the negligible volatilization loss of the solvent facilitates its industrial application.

### 3.4 Mechanism

Tetraglyme has been proved that it has good absorption of SO_2_ and regenerative capacity, due to the physical interaction between tetraglyme and SO_2_.^36,44^ As the ionic liquid exhibits better absorption and similar regeneration performance than tetraglyme, it is speculated that there is a large similarity in the absorption mechanism between them. ^1^H-NMR, ^13^C-NMR and IR are further used in the study of absorption mechanisms.

SO_2_ has the characteristics of three absorption peaks in the infrared spectrum.^45^ The antisymmetric stretching vibration peak around 1330 cm^−1^ and the bending vibration peak near 528 cm^−1^ can be seen clearly after the ionic liquid absorbs SO_2_. However, the symmetric stretching vibration peak at 1150 cm^−1^ overlaps with the position of the C–O vibration peak, thereby covering the vibration peak of SO_2_. This result is quite consistent with the infrared spectrum before and after SO_2_ absorption by tetraglyme alone. There was no significant change in the large absorption peaks of 2827 cm^−1^ to 3184 cm^−1^ before and after absorption of SO_2_, indicating that NH^+^_4_ itself had little effect on SO_2_ absorption, and the supramolecular structure of the cation still retains after absorbing SO_2_. Comparing the infrared spectrum of the ionic liquid before and after SO_2_ absorption, it can be seen that there is no significant change in the position of all absorption peaks of ionic liquid, and no new absorption peaks were produced except for the absorption peak of SO_2_. This indicates that after the ionic liquid absorbs SO_2_, there is no new chemical bond formation in the system, and the main interaction between SO_2_ and the ionic liquid is the physical interaction.

As can be seen from the ^1^H-NMR data of Fig. 2, when the tetraglyme and the ionic liquid absorb SO_2_, the chemical shifts of all hydrogen atoms move toward the lower field. After the absorption of SO_2_ by tetraglyme, the chemical shifts of hydrogen atoms move from 3.95, 3.85, 3.69 to 4.18, 4.09, 3.88, respectively. And the absorption of SO_2_ by [NH_4_-tetraglyme][SCN] ionic liquid causes the chemical shift of hydrogen atoms to move from 3.88, 3.81, 3.61 to 4.18, 4.10, 3.88, respectively. It can be seen that the two solvents have similar chemical shift changes after SO_2_ absorption and the moving directions of the two substances are consistent because the displacement change is caused by the magnetic susceptibility anisotropy due to the aromatic circulation effect of SO_2_. This means that the absorption mechanism of the SO_2_ by the tetraglyme group of the ionic liquid is similar to that of the tetraglyme absorbing SO_2_ alone, that is, the physical interaction plays a major role. Further, the chemical shift of H on NH^+^_4_ also moves, and the single peak changes to a triple peak after the absorption of SO_2_, which means that the structure of ionic liquid cations gets more rigid.

In the ^13^C-NMR spectrum shown in Fig. 3, we can see that there is no significant changes in the chemical shift of the tetraglyme group carbon atom after the ionic liquid absorbing SO_2_. However, it should be noted that after the absorption of SO_2_ by the ionic liquid, the chemical shift of carbon atoms in SCN^−^ has changed noticeable from 132.06 to 130.04. The ion–dipole interaction between SCN^−^ and SO_2_ is assumed to cause the nitrogen atom on the SCN^−^ to reduce a deshielding effect on the carbon atoms on it, leading the chemical shift of its carbon atoms to move toward the high field. The chemical shifts of other carbon atoms are not significantly changed. This indicates that the interaction that causes the ionic liquid to significantly enhance the absorption capacity of SO_2_ mainly occurs between SO_2_ and the carbon atom of the SCN^−^, rather than the carbon atoms of tetraglyme.

In summary, based on the above spectral results and by comparing SO_2_ absorption capacity of solvents with different molar ratios of tetraglyme and NH_4_SCN, it is considered that the charge transfer between SO_2_ and the cation in the ionic liquid plays a major role in the process of SO_2_ absorption by the ionic liquid, and the van der Waals force between SO_2_ and SCN^−^ also plays an important role, which make the molar absorption of ionic liquid to increase about 30% compared with that of tetraglyme.

### 3.5 Comparison of SO_2_ absorption capacity in different ionic liquids

In order to evaluate the absorption performance of this ionic liquid, we compared it with other ionic liquids, as shown in Table 1. [NH_4_-tetraglyme][SCN] exhibits satisfactory SO_2_ absorption capacity under the same conditions compared with other ionic liquids, and its ability to absorb SO_2_ exceeds that of most non-functionalized ionic liquids. And it shows excellent regeneration ability. Moreover, compared with other traditional ionic liquids and functional ionic liquids, this kind of ionic liquid has the advantages of low cost and easy synthesis, making it more conducive to practical application.

**Table 1:** Comparison of SO_2_ absorption capacity in different ionic liquids^46^

Ionic liquids	Absorption temperature (K)	Absorption Capacity at 1 bar (mol SO_2_/mol IL)
[NH_4_-tetraglyme][SCN]	293	2.73
[TMG][L]^9^	313	1.7^a^
[TMG][BF_4_]^10^	293	1.27
[BMIM][BF_4_]^10^	293	1.50
[TMG][PhO]^13^	293	2.58
[TEA][L]^14^	298	0.983
[N_2224_][dimalonate]^22^	313	1.88
[HMPY][NTf_2_]^17^	298	1.092^b^
[BMIM][OAc]^19^	298	1.91
[BMIM][MeSO_4_]^19^	298	2.11
[P_666614_][Tetz]^32^	293	3.72
[P_666614_][BenIm]^25^	293	5.75
[E_3_mim][Tetz]^31^	303	4.43
[E_0_mim][MeSO_3_]^30^	303	2.30
[E_8_mim][MeSO_3_]^30^	303	6.30
[Emim][SCN]^34^	293	2.99
PEG_150_MeDABCONTf_2_ (ref. 27)	298	4.38

a 1.2 bar.

b 1.1 bar.

## 4. Conclusion

[NH_4_-tetraglyme][SCN] ionic liquid was prepared and its absorption capacity and absorption mechanism of SO_2_ were studied. Ammonium ion forms a relatively stable supramolecular structure through ion–dipole interaction and hydrogen bonding with tetraglyme, and this supermolecule structure is the cationic portion of the ionic liquid. This ionic liquid has remarkable features such as easy to prepare, low cost, and high thermal stability. This makes it possible for the ionic liquid to be a promising candidate to be applied in desulfurization absorbing–regenerating chemical process. [NH_4_-tetraglyme][SCN] has strong absorption and desorption capacity for SO_2_ and one mol the ionic liquid can absorb about 30% more SO_2_ than tetraglyme. The results of IR and NMR experiments confirmed that the interaction mechanism between SO_2_ and the ionic liquid is physical interaction rather than chemical interaction, which makes it easier to desorb SO_2_ from the ionic liquid in the regeneration process.

## Conflicts of interest

There are no conflicts of interest to declare.

## Supplementary Material

RA-008-C8RA08991D-s001
