# Supplementary material for: Solubility of sulfur dioxide in tetraglyme-NH4SCN ionic liquid: high absorption efficiency
Source: RSC Adv. 2018 Dec 18;8(73):42116–22. doi: 10.1039/c8ra08991d (PMC9092115; doi:10.1039/c8ra08991d)
Supplement: RA-008-C8RA08991D-s001 [file RA-008-C8RA08991D-s001.pdf]

Electronic Supplementary Material (ESI) for RSC Advances.
This journal is © The Royal Society of Chemistry 2018

Supporting Information

Solubility of Sulfur Dioxide in Tetraglyme-NH_4SCN Ionic Liquid: High Absorption Efficiency

Qiang Xu[a], Wei Jiang[a], Jianbai Xiao[a], Xionghui Wei[a*]

[a] College of Chemistry and Molecular Engineering, Peking University, 100871, PR China

[*] Corresponding authors: E-mail: xhwei@pku.edu.cn

Page No.	Contents
S-2	Experimental Section.
S-3	Fig. S1 Mass spectrogram of [NH_4^+-tetraglyme][SCN] ionic liquid.
S-4	Fig. S2 ^{1}H-NMR spectra of solvents with different NH_4SCN and tetraglyme molar ratio, with $CDCl_3$ as an external reference.
S-5	Fig. S3 Thermal gravimetric analysis of tetraglyme and [NH_4^+-tetraglyme][SCN]. (At the constant temperature of 353 K)
S-6	Fig. S4 Thermal gravimetric analysis of tetraglyme and [NH_4^+-tetraglyme][SCN]. (At the constant temperature of 373 K)
S-7	Fig. S5 SO_2 absorption capacities of tetraglyme and [NH_4^+-tetraglyme][SCN] at different temperatures with the pressure of SO_2 equal to 1 bar.
S-8	Fig. S6 SO_2 absorption capacities of tetraglyme and [NH_4^+-tetraglyme][SCN] in five absorption-desorption cycles.

1. Experimental section

1.1 Spectral Measurements

MS, ^{1}H-NMR and ^{13}C-NMR and IR were all used to study the structure of the ionic liquids. ^{1}H-NMR, ^{13}C-NMR and IR were also used to study the nature of the interaction between ionic liquids and SO_2. The MS result came from Bruker Solarix XR and low resolution MS was applied to distinguish the cations. The instruments used in the ^{1}H-NMR and ^{13}C-NMR experiments were a 500 MHz Bruker Avance III spectrometer. NMR experiments were performed using an external standard method. Specifically, the sample was first injected into a custom glass tube with a size of 25 cm × 3 mm and the deuterated reagent was injected into an NMR tube with a size of 17.8 cm × 5 mm. Next, a capillary tube was inserted into the NMR tube to separate the sample from the deuteration reagent ($CDCl_3$). Infrared spectroscopy experiments were performed with a Bruker Vector 22 FT-IR spectrophotometer and analyzed with a typical thin film method. The instrument has a wavenumber range of 400 cm^{-1} to 4000 cm^{-1} with a resolution of 1 cm^{-1}.

1.2 Measurement of the the strength between tetraglyme and NH_4SCN

In Fig S4, thermogravimetry was carried out for 20 minutes at constant temperature of 373 K. Both of tetraglyme and the ionic liquid lost weight in the form of tetraglyme. The accumulative absorption heat of tetraglyme and the ionic liquid were 9.70 J and 9.29 J, respectively, and the weight loss was 2.15328 g and 0.55120 g, respectively. The molar evaporation enthalpies of the two can be calculated to be 1.00 kJ/mol of tetraglyme and 5.03 kJ/mol of the ionic liquid. Therefore, the strength between tetraglyme and NH_4SCN is 4.03 kJ/mol at the temperature of 373 K.

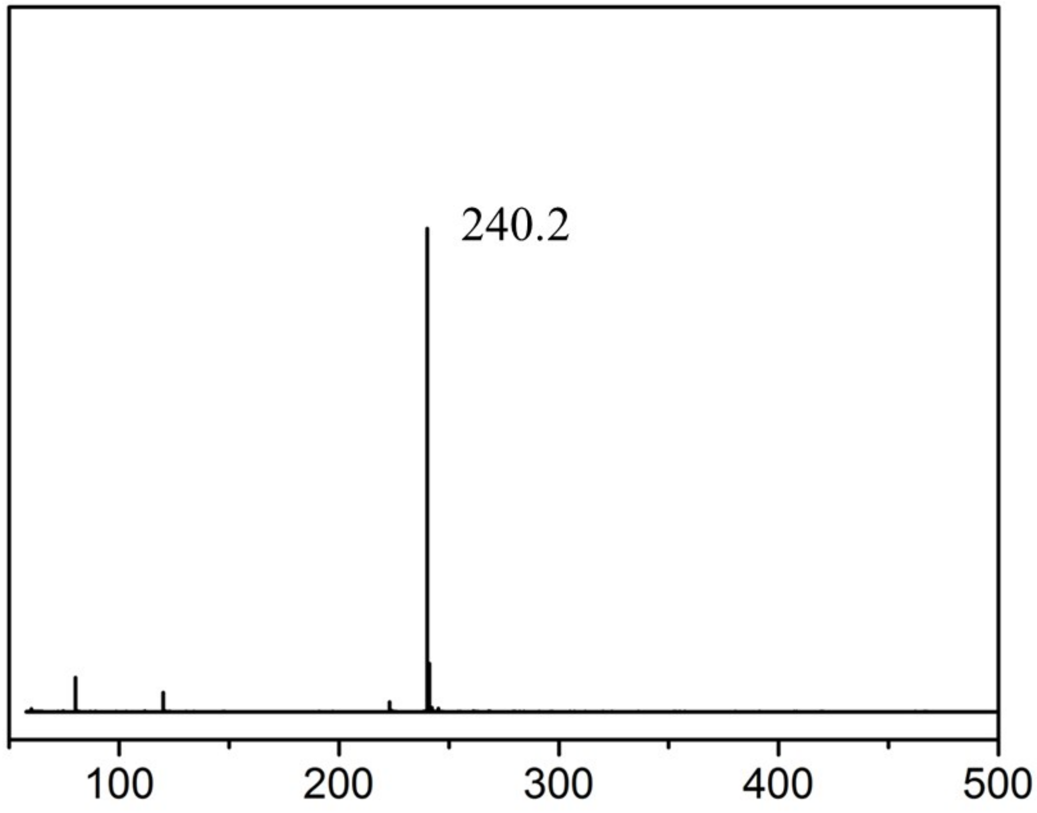

Fig. S1 Mass spectrogram of NH_4^+-tetraglyme][SCN] ionic liquid.

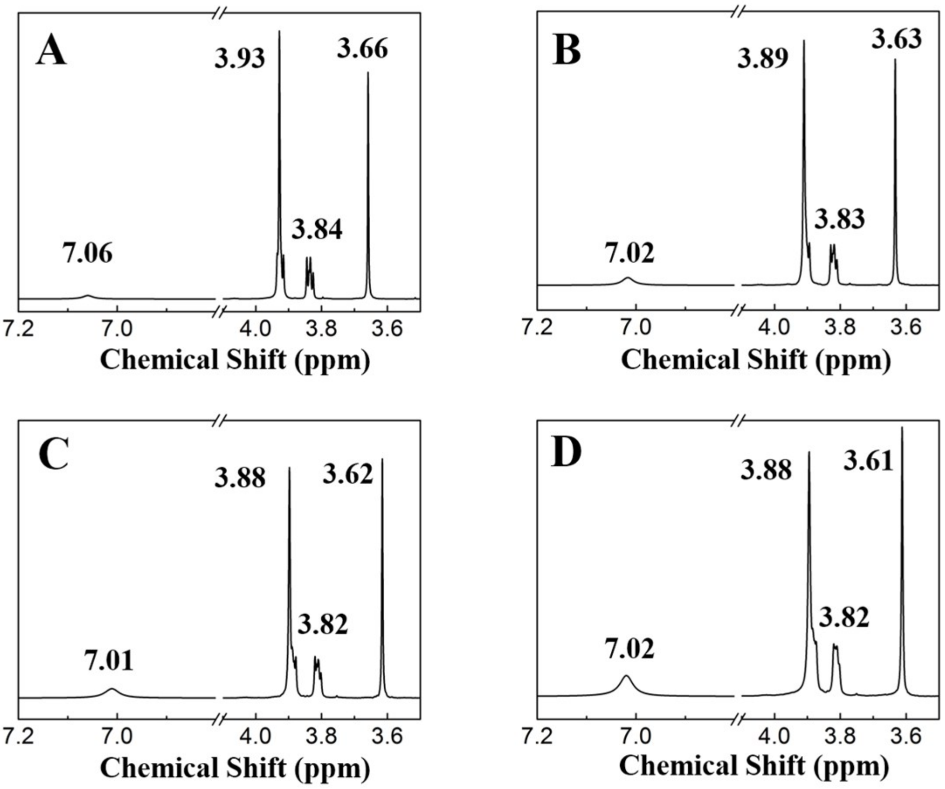

Fig. S2 ^{1}H-NMR spectra of solvents with different NH_4SCN and tetraglyme molar ratio, with $CDCl_3$ as an external reference.

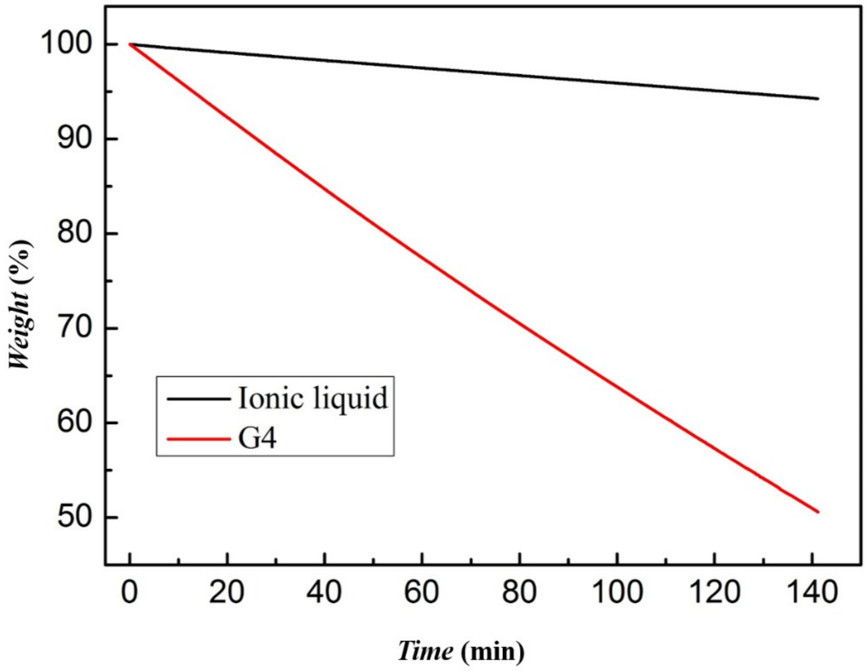

Fig. S3 Thermal gravimetric analysis of tetraglyme and [NH_4^+-tetraglyme][SCN]. (At the constant temperature of 353 K)

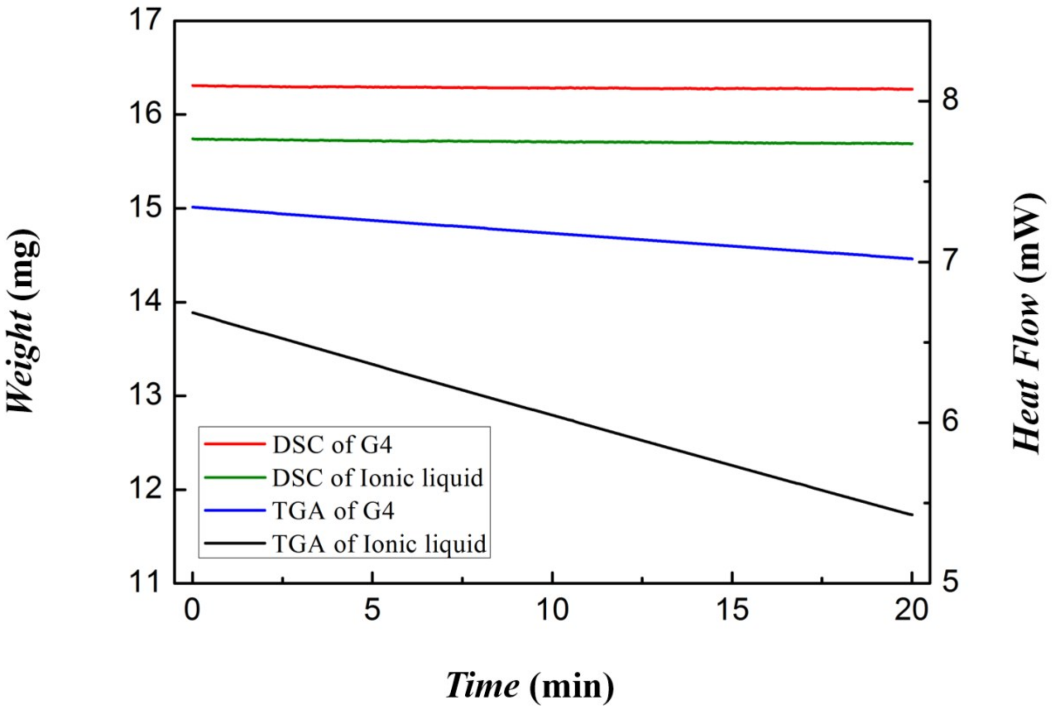

Fig. S4 Thermal gravimetric analysis of tetraglyme and [NH_4^+ tetraglyme][SCN]. (At the constant temperature of 373 K)

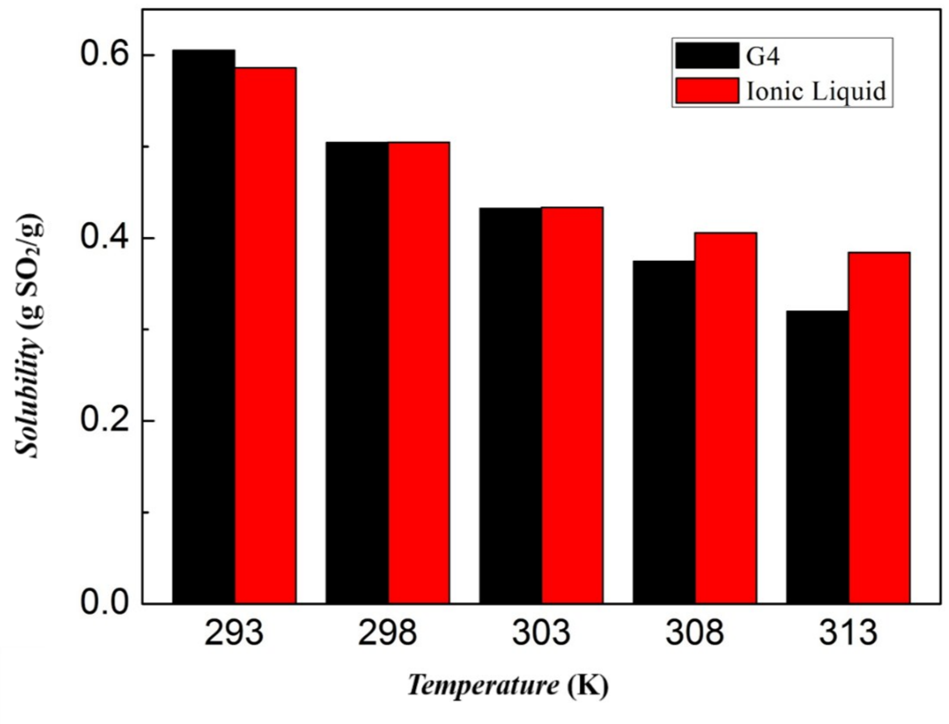

Fig. S5 SO_2 absorption capacities of tetraglyme and [NH_4^+-tetraglyme][SCN] at different temperatures with the pressure of SO_2 equal to 1 bar.

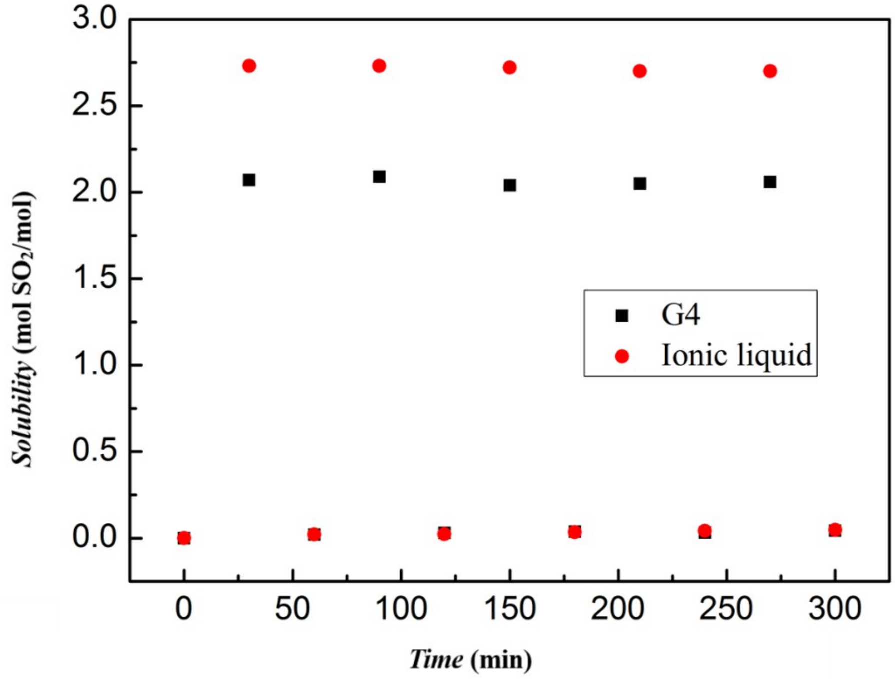

Fig. S6 SO_2 absorption capacities of tetraglyme and [NH_4^+-tetraglyme][SCN] in five absorption-desorption cycles.
